# Molecular and genetic characterization of *Cbx-Basel* , a new dominant allele of *Ultrabithorax* in D. melanogaster

**DOI:** 10.17912/micropub.biology.001321

**Published:** 2024-10-09

**Authors:** Basil Willi, Lukas Brügger, Leandra Müller, Stanley Tabor, Welcome Bender, Martin Müller

**Affiliations:** 1 Biozentrum, University of Basel, Basel, Basel-City, Switzerland; 2 Harvard Medical School, Boston, MA, USA

## Abstract

Dominant gain-of-function alleles for the homeotic gene *Ultrabithorax* ( *Ubx* ) have been known for a long time. They are summarized under the name *Contrabithorax* ( *Cbx* ). Such alleles are rather easy to spot because the morphology of the conspicuous dorsal wing appendage is often dramatically changed. The majority of these alleles is associated with chromosomal rearrangements that alter the genetic landscape of the *Ultrabithorax* locus. Thereby, UBX protein is ectopically expressed in the wing primordium where it is normally absent. Since *Ubx* specifies haltere identity, wing cells expressing UBX are determined to become haltere cells. However, apart from the prototypic allele *Cbx-1* , information on the molecular details of *Contrabithorax* alleles is scarce. Here, we present a rather detailed account on a novel Cbx-1-like allele called *Cbx-Basel* . The results of our study corroborate the model that has been postulated for the *Cbx-1* wing phenotype.

**Figure 1. Molecular and genetic analysis of Cbx-Basel:**
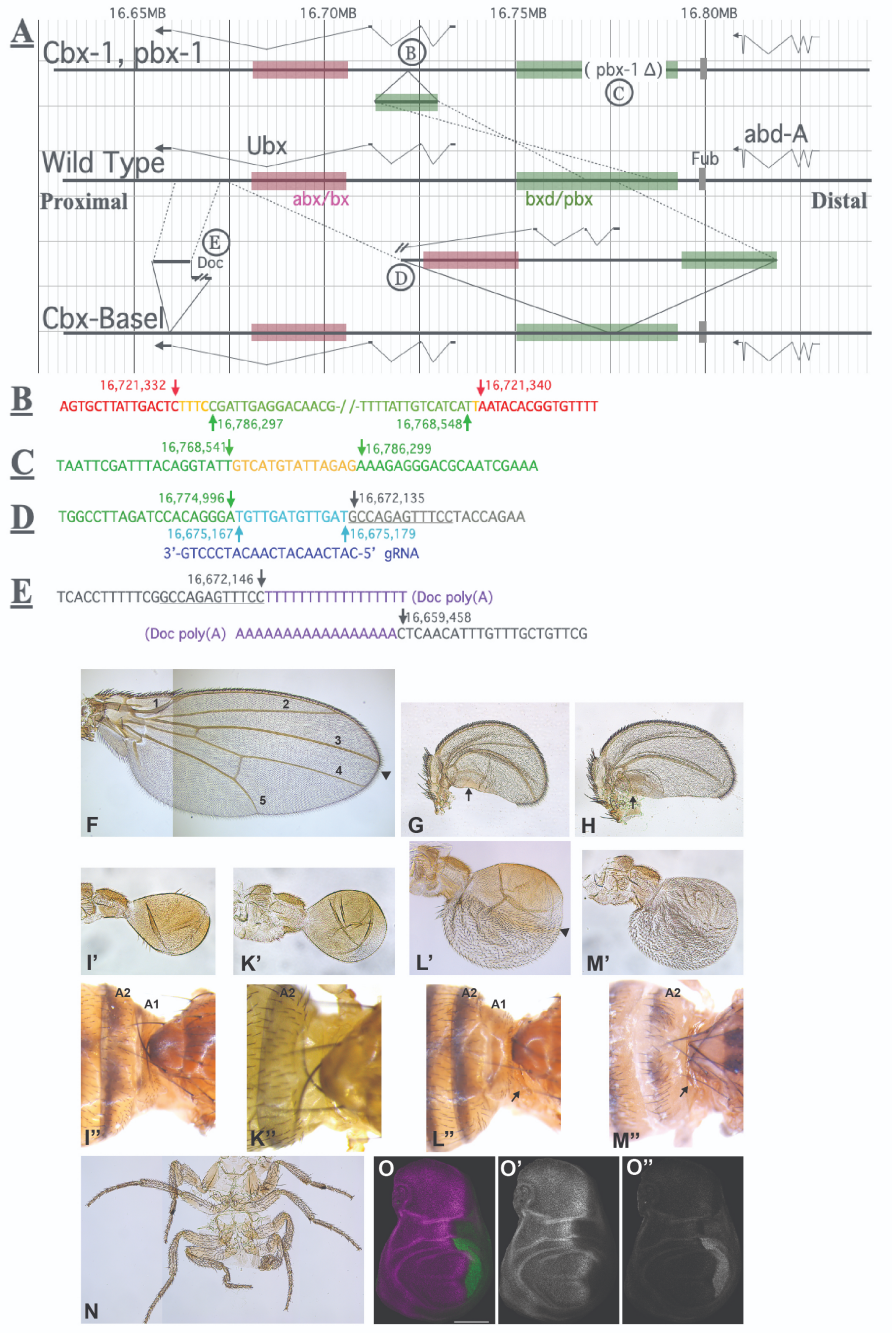
( **A** ) Organization of the *Ubx* gene in wild-type (center of panel), *Cbx-1* (top) and *Cbx-Basel* (bottom) alleles. The terms proximal and distal here and in the text indicate the position of *Ubx* on chromosome arm 3R relative to the centromere. Sequence coordinates are shown at the top according to Genome Release R6.58. The exons and introns of *Ubx* and *abd-A* are shown for each allele. On the right, the position of the *Fub* boundary (Bender and Lucas 2013) is indicated as a grey box. It demarcates the distal end of the *Ubx* domain. The realms of the *abx/bx* (in pink) and *bxd/pbx* (in green) regulatory regions are indicated. The approximate extents of these regulatory regions are based on the locations of mutant lesions (Bender et al., 1985; Peifer and Bender, 1986) and mapped parasegment-specific enhancers (Qian et al 1991; Pirrotta et al 1995 *).* At the top, the positions of the interval deleted in *Df(3R)Ubx ^pbx-1^* and the transposition present in *Tp(3;3)Ubx ^Cbx-1^* are shown. Note that in *Cbx-1* , the transposed DNA fragment is inserted in reverse orientation and that only part of the *bxd/pbx* regulatory region is translocated. Thus, in *Cbx-1* , the *abx/bx* and the transposed part of *bxd/pbx* regulatory regions are both proximal to the *Ubx* promoter *.* Organization of the *Cbx-Basel* allele is shown at the bottom of the panel. The larger tandem duplication lacks the 4 ^th^ *Ubx* exon but includes the complete *abx/bx* and much of the *bxd/pbx* regulatory regions. Consequently, the duplication inserts the *abx/bx* regulatory region into the middle of the *bxd/pbx* regulatory region. On the left, the position of a tandem 12.7 kb duplication is indicated that is associated with head-to-head *Doc* retro-transposons. Note that this duplication is about 400 bp distal to the 4 ^th^ *Ubx* exon. ( **B** ) Sequences at the transposition position of the *Cbx-1* insertion (labeled “B” in Figure 1A). Bases in red match the wild-type, those in green show the ends of the 21’750 bp transposed region, and orange bases are of unknown origin. Seven bases (AATATTA) in the 2 ^nd^ *Ubx* intron were lost in the transposition event. The insert is in reverse orientation compared to its original position. Numbers here and below indicate the wild-type map position of the bases marked with arrows (Genome Release R6.58). ( **C** ) Sequence at the *Df(3R)Ubx ^pbx-1^* break (labeled “C” in Figure 1A). Bases in green match the wild-type, and the 14 orange bases are of unknown origin. ( **D** ) Bases at the junction of the larger tandem duplication in *Cbx-Basel* (labeled “D” in Figure 1A). Green bases match the wild-type from the *bxd/pbx* region, aqua bases show a 13 base fragment from the region proximal to the *abx/bx* region, and black bases are from a slightly more proximal region of the *Ubx* transcription unit. Note that in a tandem duplication, there’s only one breakpoint. Sequencing on the distal side of the tandem duplication did not uncover any Indels. The 20 blue bases shown below were used for a guide RNA for CRISPR cutting at this duplication breakpoint (see Methods). ( **E** ) Sequences at the distal end of the smaller tandem duplication in *Cbx-Basel* (labeled “E” in Figure 1A). Black bases match the wild-type genomic sequence in the proximal region of the *Ubx* transcription unit; the underlined bases are duplicates of those underlined in Figure 1D. The purple bases represent the 3’ poly(A) stretches of *Doc* retro-transposons, which appear to be arranged head-to-head. It is not clear whether the *Docs* are intact, because the genomic sequences obtained for *Cbx-Basel* only covered small parts of them. ( **F-H** ) wings dissected from adult flies with genotype *w ^1118^* ( **F** ), *Cbx-1/+* ( **G** ) and *Cbx-Basel/+* ( **H** ) are shown to scale. Note that the wing blade of the two *Cbx* alleles is considerably smaller than that of the *w ^1118^* control. In ( **F** ), the black arrow head points to the approximate position of the anterior-posterior compartment boundary and numbers 1-5 label the five wing veins present in a normal wing. Veins 1-3 are in the anterior and 4-5 in the posterior wing compartment. Note that in the two *Cbx* alleles, only the anterior compartment with veins 1-3 is formed. The posterior compartment has collapsed because it has acquired haltere identity (arrows in ( **G** ) and ( **H** ). ( **I’, I’’** ) *Cbx-Basel/+* . ( **K’, K’’** ) *Cbx-Basel/bxd-51j* . ( **L’, L’’** ) *Cbx-Basel/pbx-1* . ( **M’, M’’** ) *Cbx-Basel/Df(3R)P10* . ( **I’-M’** ) halteres of the respective genotypes are shown to scale. *Cbx-Basel/+* ( **I’** ) and *Cbx-Basel/bxd-51j* ( **K’** ) halteres are essentially normal. Their color is brownish-yellow. ( **L’** ) *Cbx-Basel/pbx-1* halteres are clearly enlarged. The color of the posterior compartment is greyish and is covered with wing hairs. The arrow head points to the approximate position of the anterior-posterior compartment boundary These phenotypes indicate a transformation towards wing identity and are reminiscent of *pbx* loss-of-function alleles. ( **M’** ) The loss-of-function character is further enhanced in halteres of *Cbx-Basel/Df(3R)P10* flies because the anterior compartment also acquires partial wing identity. ( **I’’-M’’** ) pictures detailing the abdomen-thorax junction are shown, with anterior to the right. The first two abdominal segments are indicated as A1 (if present) and A2. In **L’’** and **M’’** , small strips of post-notal tissue are marked with arrows. ( **I’’** ) The abdomen-thorax junction is essentially normal in *Cbx-Basel/+* flies. The presence of segments A1 and A2 can be unambiguously determined by their segment-specific characteristics. ( **K’’** ) *Cbx-Basel/bxd-51j* : A1 is missing. Note that this phenotype is specific for *bxd* alleles (Bender et al. 1985). ( **L’’** ) In *Cbx-Basel/pbx-1* flies, A1 and A2 are present. Thin strips of post-notal tissue can be detected between A1 and T3. Note that this phenotype is reminiscent of weak *pbx* alleles. ( **M’’** ) *Cbx-Basel/Df(3R)P10* : A1 is missing and thin strips of post-notal tissue are present. Thus, a combination of bxd and pbx loss-of function phenotypes can be observed. ( **N** ) *Cbx-Basel/Df(3R)P10* animals frequently have 1 or 2 extra legs. Here, an example of an 8-legged male is depicted (anterior at the top). Note that like the missing A1 segment shown in K’’ and M’’, extra legs are a hallmark of *bxd* alleles. ( **O-O’’** ) immune-detection of CI and UBX ( **O** ; merge), CI( **O’** ) and UBX ( **O’’** ) protein in a wing imaginal disc dissected from a 3 ^rd^ instar *Cbx-Basel/+* larva. The disc is mounted anterior to the left. UBX is detected in a small stripe on the posterior side which is complementary to the CI pattern. Since CI immune-reactivity demarcates the anterior wing compartment, the narrow UBX stripe is located in the posterior compartment. Scale bar in O is 100 µM.

## Description

The careful genetic analysis of loss-of-function alleles isolated for the homeotic gene *Ultrabithorax* ( *Ubx* ) has shown that *Ubx* determines the identity of the posterior thorax as well as the most anterior part of the abdomen (Lewis 1978) . More specifically, *Ubx* loss-of-function alleles have phenotypic consequences in parasegmental subdivisons PS5 and PS6. Ubx expression in these two parasegments (PS) is under the control of regulatory regions *abx/bx* and *bxd/pbx* , respectively ( Figure 1A ; reviewed in Duncan 1987; Maeda and Karch 2015).

While heterozygous *Ubx ^null^ /+* flies display only a weak dominant haltere to wing transformation, homozygous flies carrying recessive lesions in the *Ubx* regulatory regions *abx/bx* and *bxd/pbx* have more spectacular adult phenotypes. In *bx/bx* flies, the anterior compartment of the third thoracic segment (belonging to PS5) is transformed to structures of the second thoracic segment (belonging to PS4). Therefore, the anterior compartment of the haltere looks wing-like and the notum is partially duplicated. In contrast, *pbx/pbx* flies transform adult structures belonging to PS6 towards PS5 identity. The most obvious consequence is the transformation of the posterior haltere towards wing identity.

Further information about the organization of the *Ubx* gene can be obtained from the analysis of gain-of-function alleles. Several of these have been isolated and are summarized under the name *Contrabithorax* ( *Cbx* ). Most of them are associated with chromosomal rearrangements and are characterized by ectopic UBX expression in the wing imaginal disc where UBX is normally absent (Bender et al 1983; White and Akam 1985; Gonzalez-Gaitan et al. 1990) . The molecular details are most complete for the prototypic allele *Cbx-1* (Bender et al. 1983) . *Cbx-1* was induced by X-irradiation, and was recovered together with the recessive *pbx-1* mutation on the same chromosome (Lewis, 1963; Figure 1A ). In *Cbx-1/+* flies, the posterior wing compartment (belonging to PS5) acquires posterior haltere identity (belonging to PS6; Figure 1G ), because UBX is ectopically expressed in the posterior compartment of the wing imaginal disc (White and Akam, 1985) . The *Cbx-1* rearrangement is associated with a transposition event that has inserted a 17’750 bp fragment ( *pbx-1 Δ* in Figure 1A ) into the second *Ubx* intron. Thereby, the distal most part of the PS6-specific *bxd/pbx* -regulatory region has been relocated. It is now present next to the *abx/bx* regulatory region and proximal to the *Ubx* promoter. According to the open for business model (Peifer et al 1987; Maeda and Karch 2015) , the relocated PS6-specific *bxd/pbx* enhancer(s) are activated one PS more anterior (in PS5), leading to the production of UBX in the posterior wing compartment (White and Akam 1985) . Consequently, the identity of the posterior wing is transformed towards haltere identity.

A new member of the *Cbx* class has been isolated and called *Cbx-Basel* (for details, see Methods). The dominant wing phenotype of *Cbx-Basel/+* flies is essentially the same as in *Cbx-1/+* (compare Figures 1G and H). One could therefore expect that in *Cbx-Basel/+* wing imaginal discs, UBX is ectopically expressed in the posterior compartment. Indeed, UBX is limited to this compartment, as shown by double staining with Cubitus interruptus (CI), a marker for the anterior compartment (Figures O-O’’).

*Cbx-Basel* is also acting as a loss-of-function *Ubx* allele. Complementation crosses with members of most *Ubx* loss-of-function classes revealed that *Cbx-Basel* belongs to the *bxd* class of alleles. There are two *bxd* -specific phenotypes: (1) loss of abdominal tergite A1, and (2) appearance of 1 or 2 extra legs from the first abdominal segment. *bxd* alleles also affect the identity of the posterior haltere, but clearly less than what is observed with *pbx* alleles (Bender et al. 1985) . All these phenotypes can be observed in hemizygous *Cbx-Basel/Df(3R)P10* flies (Figures M’, M’’ and N). They usually die as pharate adults but occasional escapers can be obtained. Trans-heterozygous *bxd51j/Cbx-Basel* flies lack the A1 tergite but don’t have extra legs (Figure K’-K’’). A weak genetic interaction is also observed in *Cbx-Basel/pbx-1* flies. It is indicated by the enlarged haltere, in which the posterior compartment is transformed towards wing identity, as well as by the appearance of thin strips of post-notal tissue (Figures L’ and L’’). The weak transformation of anterior haltere towards wing in *Cbx-Basel/Df(3R)P10* flies suggests that *Cbx-Basel* also has a weak abx/bx loss-of-function character (Figure M’).

The molecular nature of the *Cbx-Basel* allele was determined by whole genome sequencing (for details see Methods). *Cbx-Basel* is associated with a partial tandem duplication of *Ubx* ( Figure 1A ). The duplicated *Ubx* gene lacks the 4 ^th^ exon and thus lacks a large part of the Ubx protein. More importantly, the duplicated part places the intact PS5 specific *abx/bx* regulatory region next to much of the PS6 specific *bxd/pbx* regulatory region. This is analogous to the *Cbx-1* rearrangement, where part of the *bxd/pbx* (PS6) regulatory region is juxtaposed to the *abx/bx* (PS5) regulatory region. Consistent with the complementation analysis, we note that the break point falls within an interval to which intermediate and weak *bxd* alleles have been mapped (Bender et al 1985) . A second lesion in the 3 ^rd^ intron of the intact *Ubx* gene suggests a mechanism for the origin of *Cbx-Basel* . There, a tandem duplication of a 12.7 kb fragment is followed by head-to-head *Doc* retro-transposons (O’Hare et al 1991; Figure 1A ). Finally, a 53 bp deletion was detected within the tandem duplication and about 700 bp away from its distal end (sequencing data, see methods section). The very same lesion was also found in our *y w* and *y w M{vas-int.Dm}zh-2A ; M{attP}zh-86Fb* stocks. Furthermore, this deletion is also listed as a DGRP variant on FlyBase (Mackay et al 2012) . Hence, the 53 bp deletion is a polymorphism and does most likely not contribute to the Cbx-Basel phenotype. Whole genome sequencing data indicates that there are other sites of structural variation on chromosome arm 3R and elsewhere. Hence, it seems likely that the *Cbx-Basel* rearrangement arose as a consequence of multiple transposon mobilizations.

Our interpretation of the *Cbx-Basel* phenotype relies on the insertion of a PS5 ( *abx/bx* ) initiation element into the PS6 regulatory domain. Initiation elements, which respond to gap and pair rule genes in the early embryo, are sufficient to set the activity of a domain in which they reside (Iampietro et al 2010) . According to the open for business model (Maeda and Karch 2015) , the PS6 domain in *Cbx-Basel* , with all its cell type enhancers, should now be activated in PS5. *Cbx-1* gives the same phenotype, although with a complementary transposition. In *Cbx-1* , much of the PS6 domain ( *bxd/pbx* ), with its cell specific enhancers and a PS6 initiator, was moved into the PS5 domain. In both cases, the hybrid domains contain initiators for both PS5 and PS6, and the active initiator (PS5) is apparently dominant. All the cell-specific enhancers within the hybrid domain are activated in PS5 and stay active in PS6.

*Cbx-Basel* also includes an additional smaller duplication in the 3 ^rd^ *Ubx* intron, which could possibly be responsible for the phenotype. This hypothesis was tested with an attempt to resolve the tandem duplication by introducing a single CRISPR/Cas9-mediated double-strand break right at the duplication break separating *bxd/pbx* and *abx/bx* . We reasoned that intra-chromosomal recombination could delete the distal tandem duplication. If correct, revertants of the Cbx-Basel wing phenotype should be frequently recovered.

In preparation for this test, a break point specific gRNA was designed ( Figure 1D ), and a stable genomic source of this gRNA was generated by transgenesis ( *M{CbxBaselcut, w ^+^ } ^zh-86Fb^* ; see Methods for details). For the reversion test, *y w M{nosCas9, w+} ^zh-2A^* */ y w ; M{CbxBaselcut, w ^+^ } ^zh-86Fb^ Cbx-Basel / TM6C, Sb* virgins were crossed with *y w ; +* males. Non-Sb offspring that also contained the X-chromosomal *M{nosCas9, w+} ^zh-2A^* transgene were selected and wing phenotypes were scored. Reversion rates in 5 independent crosses varied from 11.2 to 28.4%, with an average of 22.3%. This observation supports our interpretation for the Cbx-Basel phenotype.

In conclusion, we have described a novel member of the *Cbx* class of dominant *Ubx* alleles. Its phenotype is remarkably similar to that observed in *Cbx-1/+* flies. In both, the wing to haltere transformation is restricted to the posterior wing compartment. The phenotypic resemblance is best explained by the fact that in *Cbx-1* and *Cbx-Basel* , the PS6-specific *bxd/pbx* enhancers become active ectopicallyin PS5 due to their juxtaposition to the *abx/bx* enhancers. It is not clear why these two regulatory regions act independently under normal circumstances. It is well documented that in the more distal part of the bithorax complex, chromosomal boundaries are separating PS-specific regulatory regions (for review see Maeda and Karch 2015). So far, attempts to localize such boundaries in the realms of the *Ubx* gene have not been successful (Ibragimov et al 2022 a,b). Based on the analysis of *Cbx-1* and *Cbx-Basel* , it seems conceivable that for proper *Ubx* function, boundary elements are not required because *abx/bx* and *bxd/pbx* are separated from each other by their target promoter.

## Methods

Fly stocks used in this study:

*Df(3R)P10/TM1* (Karch et al 1985; obtained from François Karch), *Ubx ^bx-Basel^ / T(2;3)ap ^Xa^* (Sickmann et al 2018) , *Ubx ^bxd51j^ /TM1* (RRID:BDSC_3434), *Ubx ^pbx-1^ /T(2;3)ap ^Xa^* (RRID:BDSC_3449), *Ubx ^pbx-2^ /TM1* (RRID:BDSC_8617), *Ubx ^Cbx-1^ Ubx ^pbx-1^ /TM1* (RRID:BDSC_8615), *y w M{vas-int.Dm}zh-2A* and *M{attP}zh-86Fb* (Bischof et al 2007; obtained from Johannes Bischof), *M{nosCas9, w+} ^zh-2A^* (RRID:BDSC_54591), *Ubx ^Cbx-Hm^* (RRID:BDSC_872).

Sequencing of *Tp(3;3)Ubx ^Cbx-1^* , *Df(3R)Ubx ^pbx-1^* and *Df(3R)Ubx ^pbx-2^* :

The approximate positions of the rearrangement breaks associated with these alleles have been previously reported (Bender et al 1983) . Overlapping PCR primer pairs spanning these DNA intervals were purchased (Microsynth, Balgach, CH) and genomic DNA was isolated from hemizygous flies. PCR reactions lacking a product were interpreted as discontinuities due to a chromosomal break point. Further PCR reactions with products spanning the putative break were used to confirm its presence. Such PCR products were purified and sent for sequencing (Microsynth, Balgach, CH).

The sequence at the *pbx-2* break is as follows: CCAGGAGTCCATGTAAGTGC/AGCCCATATGCCATTTATGG. The slash indicates the break point. Bases next to the break are 16’751’119 (C) and 16’773’036 (A) (indicated according to Genome Release R6.58).

Isolation of *Dp(3;3)Ubx ^Cbx-Basel^* :

While screening for transgene insertions into the *M{attP} ^zh-86Fb^* landing site (Bischof et al 2007) , one of the 23 fertile crosses yielded a single female with Cbx-like wings. The exact crossing scheme was as follows. In the first generation, a single surviving female injectee was crossed with *y w* males (♀ *y w M{vas-int.Dm} ^zh-2A^ ; M{attP} ^zh-86Fb^* x *y w ; +* ♂). Among the progeny of the F1 generation, the single female with Cbx-type wings was discovered. Based on the parental genotypes, this phenotype appeared out of the blue and could not at all be expected. In addition, the female had white eyes, suggesting that the mini-white marked transgene had not been inserted at the zh-86Fb docking site or anywhere else. Hence, assuming that the wing phenotype is linked to *Ubx* , the chromosomes on which the spontaneous mutation could have arisen are those underlined . In order to test whether the phenotype segregated according to mendelian genetics, the single Cbx-type female was crossed with *y w* males (♀ *y w M{vas-int.Dm} ^zh-2A^ / y w ; M{attP} ^zh-86Fb^ / +* x *y w ; +* ♂). In the F2, flies with Cbx-type or normal wings appeared in a 1:1 ratio, indicating that the wing phenotype observed in the F1 was dominant and heritable. In a next step, F2 males with Cbx-type wings were selected and crossed with virgins from a 3 ^rd^ chromosome balancer stock: ♂ *y w ; M{attP} ^zh-86Fb^ or + / +* x *y w ; P{S110501, w+} / TM6C, Sb* ♀. Finally, males and virgins showing the dominant Cbx and Sb markers were mated inter se and a balanced stock could be established, thereby proving linkage of the Cbx-type phenotype to the third chromosome ( *y w ; M{attP} ^zh-86Fb^ or + / TM6C, Sb* ).

The crossing scheme above indicates that the lesion giving rise to Cbx-type wings either arose on the *M{attP} ^zh-86Fb^* or *+* chromosome. Since the 3P3-RFP marker had been previously removed from the *M{attP} ^zh-86Fb^* landing site, it was impossible to distinguish between them just by inspection of the flies with a fluorescent binocular. In order to determine whether *M{attP} ^zh-86Fb^* is linked to the Cbx phenotype, seven flies from the TM6C balanced stock were analyzed by PCR with *M{attP} ^zh-86Fb^* specific primers. Apart from the control, none of them produced a PCR-product. It was concluded that most likely, a spontaneous lesion arose on the *+* chromosome originating from the *y w ; +* stock.

Genetic complementation crosses (see text) strongly indicated that the founding female fly isolated in the F1 acquired a lesion in the *Ubx* gene. Therefore, the mutation is called *Ubx ^Cbx-Basel^* or just *Cbx-Basel* .

Sequencing of *Dp(3;3)Ubx ^Cbx-Basel^* :

High molecular weight genomic DNA was prepared from *Ubx ^Cbx-Basel^ /Df(3R)P10* pharate adults. Whole genome sequencing was performed by Novogene (Sacramento, CA). Approximately 10 million reads, each of 150 bases, were aligned to the reference sequence using BWA (Li and Durbin, 2009) .

Sequence information on the 53 bp deletion: CCATTTTCACATATGGTGAC//CACCAATCAAAGCTCATTTA. The slash indicates the break point. Bases next to the break are 16’774’252 (C) and 16’774’306 (C) (indicated according to Genome Release R6.58).

Genetic characterization of *Dp(3;3)Ubx ^Cbx-Basel^* :

Once the *Dp(3;3)Ubx ^Cbx-Basel^ /TM6C, Sb* stock had been established, it was used to set up complementation crosses with a collection of characterized *Ubx* alleles ( *Ubx ^bx-Basel^* , *Ubx ^bxd51j^* , *Ubx ^pbx-1^* , *Df(3R)P10* ). Trans-heterozygous offspring was carefully inspected and phenotypes documented as follows. No genetic interaction was observed with *Ubx ^bx-Basel^* . Wings, halteres and legs were dissected and mounted in Hoyer’s medium. Pictures were taken with a Leica DM2700M microscope equipped with a Leica flexacam C3 camera. Pictures of notum/abdomen-junctions were taken with a Leica M125 binocular equipped with a Leica flexacam C3 camera.

Reversion of *Dp(3;3)Ubx ^Cbx-Basel^* by CRISPR/Cas9:

A gRNA specific for the *Ubx ^Cbx-Basel^* break point was designed with the CRISPR Optimal Target Finder tool (targetfinder.flycrispr.neuro.brown.edu). The corresponding 20 bp sequence is indicated in Figure D. The gRNA was cloned into plasmid pCFD5_w according to published procedures (Port and Boutros 2022) . The new plasmid is called pCbxBaselcut. The attB landing site present on this plasmid allowed us to isolate a transgene insert in landing site *M{attP} ^zh-86Fb^* (Bischof et al 2007) . Then, recombinants were isolated from a cross between *y w ; M{CbxBaselcut, w ^+^ } ^zh-86Fb^ + / + Cbx-Basel* females and *y w ; +* males. Recombinant males could be easily spotted thanks to concomitant presence of w ^+^ eyes and Cbx wings. A stock with genotype *y w ; M{CbxBaselcut, w ^+^ } ^zh-86Fb^ Ubx ^Cbx-Basel^ / TM6C, Sb* was established. It could be used to test the reversion rate of *Ubx ^Cbx-Basel^* if a double strand break was introduced at the duplication break point by CRISPR/Cas9.

In preparation for this test, flies had to be generated in which the following three components are combined in a fly’s germline: *M{nosCas9, w+} ^zh-2A^* , *M{CbxBaselcut, w ^+^ } ^zh-86Fb^* and *Cbx-Basel* . Therefore, the following cross was set up:

♀ *y w M{nosCas9, w+} ^zh-2A^ ; Ubx ^Cbx-Hm^ / TM6C, Sb* x ♂ *y w ; M{CbxBaselcut, w ^+^ } ^zh-86Fb^ Cbx-Basel / ap ^Xa^*

From the progeny, non-Hm, non-Xa, Sb virgins with orange eyes were selected. One would expect that in the germ line of these females, CRISPR/Cas9 induces double-strand breaks at the duplication break leading to resolution of the tandem duplication. If that is the case, non-Sb progeny with normal wings should hatch from the following cross:

♀ *y w M{nosCas9, w+} ^zh-2A^ /y w ; M{CbxBaselcut, w ^+^ } ^zh-86Fb^ Cbx-Basel / TM6C, Sb* x ♂ *y w ; +*

Five matings were set up with 5 males and 5 females per cross. Crosses were grown at 25 degrees and care was taken that cultures were not densely populated. The progeny was divided into Sb (eye color white or yellow) and non-Sb flies with orange eyes (non-Sb flies with yellow eyes were not scored because they lacked *M{nosCas9, w+} ^zh-2A^* on the X; the difference between yellow (due to *M{nosCas9, w+} ^zh-2A^* ) and orange eyed (due to *M{nosCas9, w+} ^zh-2A^* and *M{CbxBaselcut, w ^+^ } ^zh-86Fb^* flies was easy to spot). The wings of the latter were carefully inspected and grouped into 2 classes of flies: (group 1) flies with Cbx wings (no reversion) or (group 2) flies with at least 1 normal wing or 2 partially reverted wings (complete or partial reversion). The reversion rate was calculated as [(group 2) : (groups 1+2)] x 100.

Immuno-fluorescence detection:

Wing imaginal discs were dissected, fixed and stained according to standard procedures (Klein 2008) . Wing discs were mounted in Vectashield (Vector Laboratories). Primary antibodies were purchased from DSHB (α-Ci (2A1; rat monoclonal) and α-Ubx (FP3.38; mouse monoclonal). Alexa-Fluor secondary antibodies were purchased from Thermo Fisher Scientific. Pictures were taken with a Leica SP5 confocal microscope and processed with OMERO.
